# The role of imaging informatics in clinical translational research: perspectives and challenges from a US academic institution

**DOI:** 10.1186/1479-5876-10-S2-A27

**Published:** 2012-10-17

**Authors:** Brent J Liu

**Affiliations:** 1 Image Processing and Informatics Laboratory (IPILab), Department of Biomedical Engineering, Viterbi School of Engineering, University of Southern California, Los Angeles, California, 90089, USA

## 

Nearly in all clinical medicine specialties, medical images and other multi-media related data are generated and need to be distributed to points of decision. Recently, the electronic patient record (ePR) with image distribution system is gradually taking over as the method for distribution of multi-media content to the clinical environment. New challenges are accompanying its spread into other clinical fields. Particularly important are the modeling and analysis of workflow of the affected clinical disciplines as well as interface and integration issues with the image-connected electronic patient record. Although the awareness of these issues is increasing rapidly, equally important is the recognition in the professional community that more rigorous scientific methods are needed to handle the clinical system development and deployment. Furthermore, medical imaging informatics is not only based on many existing concepts, theories, terminology, and methodology derived from health informatics, but also deals with different types of data including multi-dimensional medical images, graphics, waveforms, graphics and text which are focused on the cellular, tissue, and organ systems. Accordingly, medical imaging informatics requires new concepts and new tool sets to handle these types of data. This presentation aims to first introduce the basic concepts of Medical Imaging Informatics infrastructure in both research and clinical environments including PACS, RIS, HIS, ePR, standards, databases, and system integration. This will be followed with discussions of new frontier areas of research in medical imaging informatics with some examples of clinical applications in Surgery, Neurology, Oncology, and Neuro-Rehabilitation.

